# Supplementary material for: Percutaneous transhepatic or endoscopic ultrasound-guided biliary drainage in malignant distal bile duct obstruction using a self-expanding metal stent: Study protocol for a prospective European multicenter trial (PUMa trial)
Source: PLoS One. 2022 Oct 27;17(10):e0275029. doi: 10.1371/journal.pone.0275029 (PMC9612485; doi:10.1371/journal.pone.0275029)
Supplement: S1 File — (DOCX) [file pone.0275029.s001.docx]

## **Study Centers**

1. Theresienkrankenhaus und St. Hedwig-Klinik Mannheim, Germany (PTBD)

2. München Klinik Neuperlach, Germany (EUS-BD)

3. Hospital Universitario Río Hortega, Valladolid, Spain (EUS-BD)

4. Universitätsklinikum Freiburg, Germany (PTBD)

5. Hospital Universitari de Bellvitge, Barcelona, Spain (EUS-BD)

6. Complejo Hospitalario de Navarra, Pamplona, Spain (EUS-BD)

7. Universitätsklinikum Magdeburg, Germany (EUS-BD)

8. Medizinische Hochschule Hannover, Germany (PTBD)

9. Hospital Universitario Virgen de las Nieves, Granada, Spain (EUS-BD)

10. Asklepios Klinik Barmbek, Hamburg, Germany (EUS-BD)

11. Robert-Bosch-Krankenhaus, Stuttgart, Germany (PTBD)

12. Universitätsklinikum Düsseldorf, Germany (PTBD)

13. Evangelisches Krankenhaus Düsseldorf, Germany (PTBD)

14. Katholisches Klinikum Mainz, Germany (PTBD)

15. Carl-Thiem-Klinikum Cottbus (PTBD)

16. Hospital General Universitario de Alicante (EUS-BD)
