# Supplementary material for: Percutaneous transhepatic or endoscopic ultrasound-guided biliary drainage in malignant distal bile duct obstruction using a self-expanding metal stent: Study protocol for a prospective European multicenter trial (PUMa trial)
Source: PLoS One. 2022 Oct 27;17(10):e0275029. doi: 10.1371/journal.pone.0275029 (PMC9612485; doi:10.1371/journal.pone.0275029)
Supplement: S2 File — (DOCX) [file pone.0275029.s002.docx]

**Study Protocol**

**PUMa Study Version 4.3 (08.08.2019)**

Prospective multicentre study to compare percutaneous transhepatic cholangiodrainage and ultrasound-guided bile duct puncture with endosonographic guided cholangiodrainage in malignant extrahepatic biliary duct stenosis using a self-expanding metal stent

Planned intervention:

Percutaneous transhepatic cholangiodrainage (PTBD) with (colour contrast Doppler) ultrasound-guided bile duct puncture

Endoscopic ultrasound-guided cholangial drainage (EUCD):

EUS-guided, antegrade, transpapillary biliary drainage (EUS-AD)

EUS-guided transhepatic drainage (EUS-HD)

EUS-guided common bile duct drainage (EUS-CD)

Study registration number: NCT03546049

Vote of the ethics committee

for the leading study centre in Mannheim from 6.4.2018: 2018-522N-MA

Amendment of 13.8.2019

**1. INTRODUCTION**

1.1 Scientific background

In malignant bile duct stenosis, a small percentage of patients with malignant bile duct stenosis fail to undergo endoscopic retrograde cholangiopancreaticography (ERCP). This is due to anatomical changes or a non-cannulated papilla. Percutaneous Transhepatic Cholangiodrainage (PTBD) has been the method of choice for years after unsuccessful ERCP and is widely available. However, numerous publications have reported a relatively high complication rate and 30-day mortality of up to 33% (1), making it necessary to establish a new reserve procedure for bile duct drainage. With endosonographically guided cholangiodrainage (EUCD), such a procedure has been established in recent years, since the initial description by Giovannini M in 2001 (2). In EUCD, the bile duct is punctured transmurally (through the stomach or intestinal wall) from inside the body. Four main drainage procedures are used: EUS-guided antegrade drainage via the papilla (EUS-AD), which is most similar to PTBD in principle, since it maintains the normal anatomy (bile flow via the papilla or jejunal loop in biliodigestive anastomosis), the most common EUS-guided hepaticogastrostomy (EUS-HGS), the EUS-guided choledochoduodenostomy (EUS-CDS) and the EUS-guided rendez vous procedure with ERCP (EUS-RV).

A recently published meta-analysis on percutaneous versus endosonographic bile duct drainage (3) concluded that the technical success rate of both procedures is equivalent, but that PTBD has a lower clinical success rate as well as more complications and a higher rate of re-interventions. It was therefore concluded that endosonographic bile duct drainage should be preferred to PTBD. However, the listed retrospective and prospective comparative studies either provide no information about the PTBD technique performed or use PTBD in the comparative arm in a way that does not correspond to the possibilities of this method.

The following is a commented overview of the comparative studies between PTBD and EUCD (12) (**Table 1**):

| **Author** | **Study type** | **PTBDs (n)** | **Adverse events (n)** | **Re-interventions (mean frequency)** | **Method of PTBD access** | **Special comments** |
| --- | --- | --- | --- | --- | --- | --- |
| Artifon 2012 (4) | Prospective | 12 | 3 (25%) | Not analyzed | fluoroscopic and ultrasound guidance | 4 external drainages before metal stent insertion |
| Bapaye 2013 (5) | Retrospective | 26 | 12 (46 %) | Not analyzed | fluoroscopic guidance | only 12/26 (46%) metal stents and 14/26 (54%) external drainages |
| Kashab 2015 (6) | Retrospective | 51 | 20 (39%) | 0,80 (n=41) | no detailed description | Not reported whether metal stents were used or not, many scheduled re- interventions, many bile leaks (n =17) |
| Sharaiha 2016 (7) | Retrospective | 13 | 7 (54%) | 1.70 (n=22) | no detailed description | benign and malignant bile duct obstruction were mixed, number of metal stents remains unclear |
| Lee 2016 (8) | Prospective | 32 | 10 (31%) | 0,93 (n=29) | fluoroscopic guidance | 2 step intervention: external drainage before metal stent insertion, just 15 (48%) of metal stents inserted |
| Sportes 2017 (9) | Retrospective | 20 | 2 (10%) | 1,05 (n=21) | ultrasound guidance | external drain was left after metal stent implantation and removed some days later when stent implantation was clinically successful, scheduled re-interventions were mixed with unscheduled re-interventions |

A comparative study, in which both methods are used and analysed to their best advantage, would require some conceptual changes. On the one hand, PTBD should be used in an optimal way. The following aspects should be considered (10):

1. The primary puncture of the bile duct should not be performed "blind" under radiological fluoroscopy, but under colour contrast Doppler sonographic control, in particular to avoid the puncture of intrahepatic blood vessels (11)

2. The access route of PTBD should preferably be transxyphoidal via the left lobe of the liver, since fewer complications are to be expected here than with the right access route (pain with intercostal position of the drainage, pleural complications) (12).

3. Whenever possible, metal stent-implantation should be performed in the first session and no external or internal/external plastic drainage should remain percutaneous, as this can lead to complications such as an outward or intra-abdominal bile leakage. (10).

4. Endoscopic visual inspection may be helpful in controlling stent release, as it allows precise control of the placement of the distal end of the stent in the duodenal lumen. In biliodigestive anastomosis, endoscopic control is usually not necessary because the stent usually has enough room to unfold and drain bile in the anastomosed loop of the small intestine (10).

5. A portal vein-oriented puncture of a peripheral bile duct is particularly helpful if the intrahepatic bile ducts are hardly congested (13).

The same applies to EUCD. For this, the best method and the best devices (e.g. in the choice of the metal stent) according to the current state of knowledge should be used (14). As already mentioned, various procedures are available here (transluminal stenting, antegrade stenting while retaining the existing bile duct anatomy and the rendezvous procedure with conventional ERCP). In a recent publication by Tyberg and colleagues, an algorithm was proposed to determine how the respective method should be applied depending on the dilatation of the intrahepatic bile ducts (dilated versus non-dilated) and the success of the rendezvous procedure or the success of an antegrade stenting can take place (in the next step then transluminal, e.g. as EUS-HGS or EUS- CDC) (15). Most of the studies conducted so far have only selectively chosen one of the different procedures of EUCD as a comparative method to PTBD, which could have led to a selection bias.

In contrast, the planned study will compare the three main techniques of EUCD, i.e. antegrade drainage, transhepatic drainage and drainage into the D. common bile duct, with PTBD and its optimizations described above. The specifications for the EUCD were coordinated with a recognized expert in the field of EUCD (Prof. U. Will, Gera). The study will be conducted prospectively, multicentre and non-randomized (reason for non-randomization: each centre offers the technique that it masters best).

1.2 Rational

PTBD and EUCD are recognized reserve procedures for patients with malignant biliary stenosis when ERCP was not successful or not feasible for surgical-anatomical reasons. Both procedures have a certain risk of complications. In previous studies, the technical success rate of both procedures was almost identical, whereas PTBD tended to cause a higher rate of complications, a higher number of reinterventions and a longer hospital stay. However, PTBD was presumably not performed under equivalent, optimised procedures (see 1.1.). It is expected that PTBD in this optimised procedure causes fewer complications and fewer re-interventions are necessary than shown in previous studies. In addition, the occurrence of intervention-related pain is analysed in a standardised manner using a visual analogue scale (18).

On the other hand, the EUCD is a dynamically evolving procedure whose technical equipment and therapeutic algorithms are constantly evolving (19). The planned multicentre study should therefore show how both procedures compare under equivalent conditions and according to current standards in selected centres in Germany. In particular, the success rates, but also the complication and reintervention rates are to be determined, so that finally, if necessary, it can be reformulated which of the two methods would be preferable in future as the primary reserve method according to ERCP.

1.3 Risk-benefit assessment

At the respective study centre, patients with malignant bile duct stenosis are prospectively offered the method that is best mastered there. Either PTBD or EUCD. This way, no additional risk beyond the usual intervention-related risk is incurred. With presumably similar technical and clinical success, the study should show to what extent the higher complication rate, higher rate of reintervention and longer hospital stay attributed to PTBD actually apply. On the other hand, the study offers the opportunity to make the EUCD method and its possibilities and risks even better known.

**2. OBJECTIVES AND ENDPOINTS**

Primary objective and primary endpoint

The primary objective of the study is to show that PTBD is not inferior to EUCD in terms of technical success rate. The technical success rate is defined as the successful implantation of a metal stent into the bile duct system (= successful drainage of the contrast medium via the metal stent within one minute after injection).

The primarily technical success of the intervention is decisive for the further procedure following the examination (reintervention necessary ? surgery necessary ?) and is presumably almost identical to the clinical success of the intervention, which can only be verified after 7 days (drop in bilirubin value ≥ 50%). The technical success rate was therefore preferred to the clinical success rate as the primary endpoint.

In PTBD there is only one possibility of metal stenosis insertion across the tumor stenosis in the common bile duct (blunt). In EUCD, the additional option of transluminal stent placement in the context of EUS-HGS or EUS-CDS remains.

The prompt (1 week) implantation of a metal stent after the application of an external drainage in PTBD is also considered a technical success. However, the application of an external drainage is counted as "reinvention".

Secondary objectives and secondary endpoints

In addition to the technical success rate, both interventions will be compared descriptively with regard to other endpoints. All endpoints or points in time refer to the technically successful intervention.

1. Clinical success rate of bile extraction, defined as reduction of the bilirubin value of ≥ 50% 7 days after technically successful intervention in relation to the baseline value before intervention.

2. The occurrence of at least one complication (including death) within 30 days (description + count)

3. Duration of bile duct intervention (PTBD/EUCD) in minutes

- for PTBD: from the application of the local anaesthetic to the application of the skin dressing

- for the EUCD: from the insertion of the endoscope to the removal of the endoscope

4. Post-interventional pain sensation on day 1 and day 7 after intervention (in relation to baseline before intervention): visual analogue scale

5. Length of hospital stay (from the day of the first intervention to the day of discharge) in days

6. Number of re-interventions between the first intervention and the end of the follow-up

(n) after 6 months.

7. Overall survival (overall survival including any cause of death) and disease specific survival (overall survival which only includes the underlying malignant disease that led to bile duct obstruction)

**3. STUDY CONCEPTION/ DESIGN**

3.1 Study design

Prospective, multicentre, non-randomized, open, confirmatory, 2-arm non-inferiority study in parallel group design

3.2 Duration and timetable

The duration of the clinical trial for the individual participant is 6 months follow-up time after intervention.

The total duration of the clinical trial is expected to be 36 months. The recruitment of patients will start on 1.12.2018.

Total duration:

Duration of the clinical phase: [36 months].

[30 months]

FSI (first subject in): [1.12.2018]

LSI (last subject in): [1.12.2020]

LSO (last subject out): [31.5.201]

DBL (database lock): [30.06.2020]

Completion of statistical analysis: [1.10.2020]

Final study report: [1.12.2021]

**4. PATIENT AND CENTRE SELECTION**

4.1 Number of patients

According to the case number calculation (Chapter 9.1), 212 patients, i.e. 106 patients per intervention group, are needed to achieve a power of 80% under the assumptions made. Since this is a non-randomised study, in which only one intervention is offered within a centre, recruitment should not be terminated until at least 106 patients have been enrolled in both arms. If the centres belonging to the same intervention arm have together reached the recruitment target of 106 patients, but the centres of the other arm have not yet reached it, recruitment may continue.

The recruitment and treatment of patients will be carried out in the respective study centres. Each centre participating in the study should be able to enrol at least 10 patients within the planned recruitment period of 24 months.

If the total number of cases is not reached by the planned recruitment end (31.01.2020), the recruitment period may be extended if necessary. If recruitment is accelerated, recruitment will be terminated earlier accordingly.

4.2 Trial centres

The present study is conducted multicentre (5 centres PTBD, 5 centres EUCD).

Due to the complexity of the interventions, only one of the two interventions is carried out in each centre, namely the one that is also carried out there by default.

When selecting the centres for the study, care is taken to ensure that the same number of centres participate for both intervention groups.

4.3 General criteria for patient selection

No patient may be enrolled in the clinical trial more than once.

4.4 Inclusion criteria

Patient is at the age of ≥ 18 years with inoperable, malignant underlying disease and shows a clinically relevant stenosis of the common bile duct (= subhilar). An ERCP was not successful or could not be performed due to surgically induced anatomical changes

Laboratory chemical, at least twofold increase in the total bilirubin value of: ≥ 2.0 mg/dl

The malignant underlying disease was histologically confirmed

An abdominal sonography was performed

A further sectional imaging of the abdomen is available in order to rule out, in addition to planning the intervention, an advanced underlying disease with a severely limited prognosis (e.g. metastatic liver):

CT abdomen or MRT abdomen

Written consent has been obtained

4.5 Exclusion criteria

Individuals who meet one of the following criteria will not be enrolled in the clinical trial:

There is limited blood clotting (Quick < 50%, PTT > 50 sec., platelet count > 50/nl)

There is an extrahepatic bile duct carcinoma of the Klatskin type in stage bismuth 2-4 or an intrahepatic bile duct carcinoma

The malignant underlying disease is operable or can be cured by chemotherapy (e.g. aggressive non-Hodgkin's lymphoma)

The patient is pregnant or breastfeeding

The patient participates in another interventional study on EUCD or PTBD

4.6 Termination criteria

4.6.1 Premature discontinuation of individual patients

Every patient has the right to withdraw his or her consent to participate in this clinical trial at any time without giving reasons. If possible, the time and reason for the termination of the study should be noted in the CRF. Data collected until then will also be noted in the CRF.

Reasons for the premature termination of the study may be

- withdrawal of consent by the patient.

- Study participants can no longer be contacted.

4.6.2 Premature termination of the trial or closure of a centre

In the event of an accumulation of serious adverse events (SUEs) (see Chapter 8.2) in a trial site or trial arm (PTBD or EUCD), the Steering Committee is involved. The Steering Committee reserves the right to draw the appropriate consequences from the reports received, such as the closure of a trial site or termination of the study.

A serious adverse event (SUE) is any adverse event that occurs during the course of the study, regardless of the link to the intervention, and has one of the following consequences for the patient

- Death

- Unplanned surgical intervention necessary

- Permanent damage or permanent disability remains

- Unscheduled return to hospital or extended hospital stay: > 10 days

- Unplanned stay intensive care unit: > 1 night

All investigators involved must be informed immediately of any stop or interruption of the trial. The decision is binding on all trial centres and investigators.

If the clinical trial is terminated prematurely, all study materials (completed, partially completed) must be returned to Data Management.

**5. TREATMENTS/ INTERVENTIONS**

5.1 Description of the treatment(s)/ intervention(s)

PTBD

PTBD is performed in the first step by means of (colour contrast Doppler) ultrasound-guided puncture of an (dilated) intrahepatic bile duct using an access needle (e.g. Chiba needle (22G/0.7mm diameter). In the next step, the application of contrast medium into the bile duct system is performed under radiological fluoroscopy. The access route should be at a sufficient safety distance from neighbouring structures, i.e. the pleura in case of right-sided access.

Ideally, after KM filling of the bile duct system, a wire should be advanced distally beyond the stenosis in the common bile duct. This can also be done after changing to a larger wire (from 0.018" to 0.035"). After placement of an appropriate guide wire, a corresponding metal stenosis implantation (SEMS) should be performed in the same session in the area of the stenosis of the common bile duct (usually 8 or 10 mm diameter with different lengths (usually 6 -10 cm).

For further precision, the release of the metal stent can (not obligatory) be controlled by an additional luminal endoscopic visualization. (usually in relation to the papilla or the protrusion into the duodenum). This is particularly relevant with regard to endoscopic reintervention. Even in cases of tumour-related duodenal stenosis (use of a thinner endoscope) or altered anatomy after abdominal surgery (e.g. access via the feeding loop after Billroth II surgery). it is very often possible to reach the papilla (e.g. using a gastroscope).

Leaving a percutaneous plastic drainage after successful implantation of a metal stent should be avoided (counted as reintervention). If metal stent implantation is not possible in the initial PTBD, an internal/external plastic drainage should be inserted and the sole external drainage should be avoided (highest risk of complications).

EUCD

Endosonographically controlled cholangiodrainage (EUCD) is performed according to the current state of knowledge of this technique (2-9,14,15).

The choice of the respective EUCD method depends on the examiner. First and foremost, however, an antegrade stenting in the sense of an EUS-AD (wire is passed over the stenosis) should be performed.

should be attempted, since - in contrast to the transluminal procedure - the original anatomy of the bile duct system is preserved and the least complications are expected.

In the event of unsuccessful wire advancement via the tumor stenosis in the context of an intended EUS-AD, the next step should be an EUS-guided transhepatic drainage (EUS-HD) with metal stent implantation or EUS-guided choledochal drainage (EUS-CD) with metal stent implantation. The respective access route - transesophageal, transgastral, transduodenal or transenteral - depends on the disease or anatomy (e.g. in the case of Roux Y anastomosis transenteral). In the evaluation of this study, the respective, access-path dependent interventions are subsumed under the designations of an EUS-HD or an EUS-CD.

EUS-guided interventions such as the EUS-guided rendezvous procedure, EUS-guided cholecystomy or EUS-guided jejunostomy, which is used e.g. in afferent loop syndrome and usually requires the use of a lumen apposing metal stent (LAMS), are not the subject of this study.

5.2 Risks of the study intervention

The study does not pose any additional risk for the patient in addition to the usual risks of the respective intervention.

5.3 Randomisation and blinding

Randomisation is not carried out, as one centre cannot usually offer both interventions (PTBD and EUCD) of the same high quality due to the complexity and experience required. Therefore, each centre offers the respective standard method (EUCD or PTBD). At least 10 study centres will participate in the study. When selecting the centres, care is taken to ensure that both university hospitals and non-university institutions are selected for both groups. All potentially eligible patients will be screened (see Chapter 6.1) and, if suitable, informed about the study. It is assumed that the recruited patients of both groups have similar characteristics and are comparable. Possible differences in patient characteristics are taken into account in the data evaluation. However, it is not expected that different patient characteristics (e.g. pancreatic carcinoma and bile duct obstruction versus LK metastasis in liver hilus with bile duct obstruction) will have a relevant influence on the success or failure of the intervention.

Due to the nature of the interventions, the attending physician cannot be blinded. Since only one of the two surgical techniques is used in a centre, the patient and study nurses cannot be blinded either. With the exception of the secondary endpoint of pain perception, the endpoints, especially the primary endpoint, are objectively measurable outcomes. Therefore, it is assumed here that there is little room for influence and that the risk of bias is low. The biometrician has no access to the data until after the database has been closed.

5.4 Co-interventions

After the clinically successful intervention on the bile duct (decrease of the bilirubin value), palliative chemotherapy can be performed depending on the underlying malignant disease. The respective chemotherapy is specified in the documentation. Palliative chemotherapy can influence the rate of reintervention (usually reduction of the rate of reintervention), disease-specific survival (usually prolongation) and overall survival (usually prolongation). This is taken into account in the evaluation of the data.

5.5 Re-interventions

Re-interventions are defined as renewed or repeated performance of PTBD or EUCD. Re-interventions may become necessary to complete a technically successful implantation of a metal stent in the bile duct. This may be the case, for example, if in the case of PTBD only the creation of an external drainage (drainage to the outside, no overcoming of the tumor stenosis) was possible in the first intervention. Planned re-interventions are avoided within the study concept (e.g. leaving the external/internal drainage after technically successful PTBD with stent implantation and radiological control of the result on the following day). After technically successful implantation of the metal stent, stent closure, stent migration (change of position) or other stent malfunction may occur in the further course (following months). This is usually clinically visible by an increase in the laboratory chemical parameters for bile duct occlusion (increase in bilirubin, increase in alkaline phosphatase) or by cholangitis (additional signs of infection). Any Re-intervention on the bile duct that is related to the primary method used, PTBD or EUCD, is considered as re-intervention in the documentation of the data. Thus, an operation (for example: creation of a biliodigestive anastomosis) which becomes necessary as a result of the "malfunction" of the metal stent is also counted as a re-intervention (!).

5.6 Pre- and concomitant diseases

Pre- and concomitant diseases besides the underlying malignant disease that are present at the time of informed consent are not documented, as they are not expected to have a relevant impact on key endpoints of the study such as the technical and/or clinical success rate or the occurrence of complications of the intervention. By differentiating between Overall Survival and Disease Specific Survival, it is possible to specify the proportion of disease-specific causes (related to the underlying malignant disease causing the biliary obstruction) in the death of patients. It is expected that the underlying malignant disease is the main life-limiting factor in the observation period.

5.7 Emergency treatment

During and after the clinical trial, the investigator must ensure that, in the case of UEs, the trial subjects always receive adequate medical care.

**6. DESCRIPTION OF THE STUDY VISITS**

For the individual patient, the duration of the study consists of an intervention (PTBD or EUCD) followed by an observation period of 6 months. Before the treatment phase, there is a screening phase with examination of the inclusion and exclusion criteria.

Overview of the study visits and the data to be collected (Fig 1)


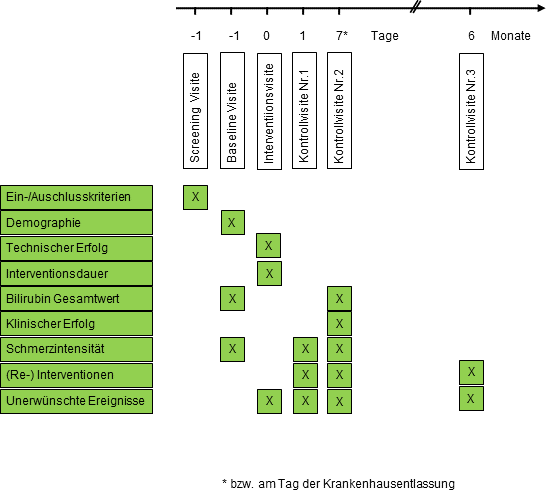


6.1 Screening visit

A paper-bound explanation of the study (own form) for the intervention (commercial form) and the sedation or anaesthesia (commercial form) of the patient is provided. Each potential study patient will receive a CT or MRI abdomen in addition to an abdominal sonography. The inclusion and exclusion criteria are checked (Case Report Form , p.2). Every patient who has been positively screened is included in the study if written consent is obtained (Case Report Form, p. 1). In the case of patients who are still capable of giving birth, a possible pregnancy is excluded by pregnancy tests.

It is assumed that almost all positively "screened" patients want to undergo an intervention under study conditions. In this situation, surgery is usually either not possible (pre-operated abdomen, advanced tumour disease) or is associated with a considerable risk. The performance of a PTBD/EUCD is therefore mostly without alternative. The screening list lists (separate form) which patients have been screened positively (inclusion and exclusion criteria fulfilled). Should a patient not be included in the study (e.g. because of refusal to participate in the study), this is briefly explained.

6.2 Baseline visit

Facts of the clinical picture are documented (Case Report Form,p.3) . Cause of malignant bile duct obstruction, date of first diagnosis of the malignant underlying disease, cause of unsuccessful or for anatomical reasons not feasible ERCP.

The bilirubin value in mg/dl as an expression of the bile duct obstruction or cholestasis is measured and noted. The baseline visit should be performed the day before the intervention. Depending on the timing within the centre, baseline visit can be on the same day as screening visit if the intervention is scheduled for the following day.

Data that are documented as part of the study:

Demographics

- Gender

- Age

- Size

- Weight

Cause of the malignant bile duct obstruction (no multiple answers)

- Pancreatic carcinoma

- Distal bile duct carcinoma or extrahepatic bile duct carcinoma (Bismuth I)

- Gall bladder carcinoma

- Stomach cancer

- Duodenal carcinoma

- Papillary carcinoma

- Metastasis of another malign disease

- Other cause

Date of first diagnosis of the underlying malignant disease

Cause of unsuccessful or for anatomical reasons not feasible ERCP (no multiple answers)

- Gastric outlet stenosis/duodenal stenosis (tumour-related or benign cause)

- Difficult to probe the optic disc using ERCP

- For (partial) gastrectomy

- Biliodigestive anastomosis

- Following bariatric surgery

- Other cause

Laboratory chemical result of bilirubin determination (in g/dl) Pain intensity on the visual analogue scale (0-10)

6.3 Treatment rounds (intervention day)

Characterisation of the respective intervention: PTBD or EUCD (Case Report Form, p.4). The duration of the intervention is noted in minutes, the technical success with "yes/no". Adverse events (description, classification into light, medium, severe and fatal) are documented.

Data documented within the study:

Date of the intervention Start of the intervention

PTBD: Start with skin anaesthesia

EUCD: Start of endoscope insertion End of intervention PTBD: Completion of skin dressing EUCD: Removal of endoscope from patient Dimensions of metal stent

Wrapping of the metal stent Intervention technically successful

PTBD/EUCD: Contrast medium outflow via the metal stent (max. 1min. after injection)

PTBD Localization

PTBD with metal stent implantation

Number of PTBDs before metal stent implantation

PTBD with metal stent release under endoscopic control

EUS-controlled, antegrade drainage (EUS-AD)

EUS-guided transhepatic drainage (EUS-HD)

EUS-guided choledochal drainage (EUS-CD)

6.4 Control visits

On the day after the technically successful intervention, the patient's pain sensation is recorded by means of a visual analogue scale (0-10) (Case Report Form; Control Visit No.1, p.5).

The next visit is on day 7 after the technically successful intervention (Case Report Form; Control Visit No.2, p.6). If a re-intervention is necessary (see Chapter 2.1), the count is only made from the day of successful intervention (metal stent implantation). If the patient is discharged from the hospital within the seven days after technically successful intervention, it must be ensured that the attending general practitioner carries out the laboratory check and the result is obtained from the general practitioner. The determination of bilirubin has been so standardized for many years that no central laboratory is necessary. In addition, the intensity of pain according to the visual analogue scale (0-10) must be obtained from the investigating physician or his representative by telephone or post on day 7 after a technically successful intervention. Adverse events and re-interventions, including the patient's discharge day (which is counted), are documented to determine the length of hospital stay in days.

The patient is called to a staging by means of CT or MRI abdomen after three (no data collection here) and six months of the technically successful intervention as an outpatient or inpatient (not study related). The date six months after intervention should be used for a detailed study visit. Previously undocumented complications in a 30-day interval and reinterventions within the six months after the intervention will be documented (Case Report Form; Control Visit No. 3, pp.7-8). If the patient is not examined at the trial centre, it must be ensured that the relevant data on re-interventions or complications can be obtained by calling the family doctor or patient.

If the patient should be referred to tumor therapy (palliative chemotherapy, immunotherapy, antibody therapy) as part of the malignant underlying disease, this will be documented with "yes/no" and the therapy will be named. It can be assumed that a tumor therapy has a positive influence on the re-intervention rate as well as on the disease-specific survival and overall survival.

Data documented in the study

Control visit no.1 (1 day after intervention)

Intensity of pain on the visual analogue scale (0-10) Complication(s) since intervention (yes/no)

Serious adverse events (SUE) since intervention (yes/no)

Control visit no.2 (7 days after technically successful intervention

Intensity of pain on the visual analogue scale (0-10) Date Bilirubin determination (after initial intervention)

Laboratory chemical result of the bilirubin determination (after initial intervention, in mg/dl)

Duration of hospital stay from day of intervention (inclusive) to day of discharge (date of hospital discharge, if later than 7 days, please add later!)

Re-intervention(s) since control visit 1 (yes/no) Complication(s) since control visit 1 (yes/no)

Serious adverse events (SUE) since intervention (yes/no)

Control visit no.3 (6 months after intervention) and/or study completion visit

The final study visit coincides with the follow-up visit after six months. To calculate the Overall Survival or Disease Specific Survival, it is noted whether the patient is still alive or when he/she died and why (date of death, calculation of survival time in days, cause of death). If the patient is not examined at the trial centre or has died in the meantime, it must be ensured that the relevant data on re-intervention, adverse events, date of death and cause of death can be obtained by calling the family doctor or relatives.

Data that are documented within the framework of the study:

Date of end of study/last contact

Reason for termination:

- Regular termination of the study (yes/no)

- Patient has died (yes/no)

Date of death

Cause of death on the ground of the underlying malignant disease (yes/no)

Cause of death other than underlying malignant disease (yes/no)

Cause of death unclear (yes/no)

Revocation of consent (yes/no)

Patient no longer appeared/follow-up data no longer to be collected (yes/no)

Intervention not successful, no new attempt

Other (specification)

Chemotherapy carried out during the course (yes/no)

Chemotherapy protocol (name): first line/second line/third line (multiple answers possible)

- Gemcitabine mono (yes/no)

- Gemcitabine/Nab-paclitaxel (yes/no)

- FOLFIRINOX (yes/no)

- Cisplatin/Gemcitabine (yes/no)

- FLO (T) (yes/no)

- FOLFIRI (yes/no)

- Paclitaxel/Ramucirumab (yes/no)

- Ramucirumab mono (yes/no)

- 5 FU mono (yes/no)

- Other chemotherapy/immunotherapy (yes/no)

Other therapy (but no chemotherapy) performed (yes/no)

**7. METHODS OF DATA COLLECTION**

The parameters that are collected and documented during the respective study visits are already described in Chapter 6.

7.1 Safety parameters

Adverse events:

Definition see 8.1.1

Adverse events are queried each time the investigator and trial subject come into contact with each other.

7.2 Efficacy parameters

7.2.1 Technical success of the intervention

Defined by the successful implantation of the metal stent at the respective examination-dependent position

In PTBD:

trans stenotic metal stent implantation, verified by the successful outflow of the contrast medium via the metal stent into the distal intestinal segment (imaging: radiological fluoroscopy; this is not study related)

In EUS-AD:

trans stenotic metal stent implantation, verified by the successful outflow of the contrast medium via the metal stent into the distal intestinal segment (imaging: radiological fluoroscopy; this is not study related)

In EUS-HD:

Metal stent implantation transhepatic, verified by the successful outflow of the contrast medium via the metal stent into the intrahepatic dilated bile ducts (imaging: radiological fluoroscopy; this is not study related)

In EUS-CD:

Metal stent implantation transduodenal into the common bile duct, verified by the successful outflow of the contrast medium via the metal stent into the dilated common bile duct (imaging: radiological fluoroscopy; this is not study related)

7.2.2 Clinical success of the intervention

Defined as a drop in bilirubin value in mg/dl ≥ 50% 7 days after completed intervention in relation to the baseline value before intervention

7.2.3 Overall survival

Defined as the time from the beginning of the intervention until death, for whatever reason. If there is no more contact with the patient ("lost to follow up") or the patient is still alive after 6 months, the overall survival is censored to the last contact.

7.2.4 Disease Specific Survival

Defined as the time from the beginning of the intervention until death caused by the underlying malignant disease. If there is no more contact with the patient ("lost to follow up") or the patient is still alive after 6 months, the overall survival is censored to the last contact.

**8. ASSESSMENT OF PATIENT SAFETY**

8.1 Definition and documentation of complications

In the study population with advanced oncological diseases, numerous tumour- and tumour therapy-related adverse events are to be expected.

For the assessment of patient safety with regard to the interventions performed in the study, only certain adverse events in terms of potential complications of PTBD/EUCD, of which a major part is to be expected within a period of 30 days after the intervention, are of interest and complications are to be documented in the notification form.

The following potential complications of PTBD and/or EUCD are recorded in the CRF with date and severity (mild, moderate, severe, fatal) (see Table 1, below) (multiple entries possible):

- Bleeding/haematoma

- Haemobilia

- intra-abdominal bile leakage

- Pneumothorax

- Biliary pleural effusion

- Pneumoperitoneum

- Perforation of the gastrointestinal tract

- Therapy-relevant (cholangial-)sepsis

- Newly occurring cholangitis (see comment below)

- Abscess

- Purulent peritonitis

- Cholecystitis

- Metal stent dysfunction/migration

- Other (description in free text)

To be distinguished from the above mentioned complications and not to be recorded:

- metal stent-closure caused by the ingrowth of tumour tissue or by choledocholithiasis

- Adverse events associated with anaesthesia and/or sedation

- Adverse events associated with chemotherapy

- Cholangitis as a possible complication of the intervention by PTBD or EUCD requires a special consideration: Only the newly appeared and not the pre-existing cholangitis is considered a complication ! Since the patients are treated peri-procedurally prophylactically with antibiotics, cholangitis is only considered a complication if an intensification of antibiotic therapy becomes necessary and the cholangitis can be safely attributed to the intervention !

- Postinterventional pain (will be collected separately)

Severity of the complication

Table 1: Severity of complications according to the recommendations of the American Society for Gastrointestinal Endoscopy (ASGE) (16,17)

| **Severity of the complication** | **Slightly** | **Moderate** | **Severe** | **Fatal** |
| --- | --- | --- | --- | --- |
| Procedure aborted or not yet started because of UE | x |  |  |  |
| Unscheduled anesthesiological measure required in connection with a procedural adverse event (e.g. endotracheal intubation) |  | x |  |  |
| Unplanned return to hospital or extended hospital stay: ≤ 3 days | x |  |  |  |
| Unscheduled return to hospital or extended hospital stay: 4-10 days |  | x |  |  |
| Unscheduled return to hospital or extended hospital stay: > 10 days |  |  | x |  |
| Unplanned stay intensive care unit: ≤ 1 night |  | x |  |  |
| Unplanned stay intensive care unit: > 1 night |  |  | x |  |
| Blood transfusion necessary (EK) |  | x |  |  |
| Additional endoscopic examination necessary (e.g. hemostasis) |  | x |  |  |
| Additional radiological intervention necessary (e.g. coiling) |  | x |  |  |
| Surgical intervention necessary |  |  | x |  |
| Permanent damage/permanent disability remains |  |  | x |  |
| Death |  |  |  | x |

8.2 Causal link between intervention and complication

As a rule, the connection or non-connection of a complication with the intervention performed is obvious. Nevertheless, a distinction is made between safe, probable and unlikely.

8.3 Outcome of the complication

Complications are treated with all available medical means in the interest of patient welfare. However, the outcome of the complication is of secondary importance in the evaluation of this study and is therefore not documented separately (exception: SUE, see 8.4). The severity of the complication sufficiently implies the need for appropriate treatment.

8.4 Definition, reporting and documentation of a serious adverse event (SUE)

Regardless of the documentation of serious or fatal complications, serious adverse events (SUE) must be documented and reported separately.

A serious adverse event (SUE) is any adverse event within the meaning of the serious or fatal complications mentioned in 8.1 that occurs during the course of the study, regardless of the link to the intervention, and has one of the following consequences for the patient

- Death

- Unplanned surgical intervention necessary

- Permanent damage or permanent disability remains

- Unscheduled return to hospital or extended hospital stay: > 10 days

- Unplanned stay intensive care unit: > 1 night

All SUEs must be documented in detail on a special documentation form (notification form "serious adverse event") and reported by fax to the lead study centre in Mannheim within 24 hours, but at least on the next working day after the notification:

Principal investigator (LKP)

University Hospital Heidelberg/Theresienkrankenhaus and St. Hedwigsklinik GmbH

(teaching hospital)

Medical Clinic 1 : Gastroenterology, Oncology and Diabetology

Senior consultant Dr. med. Daniel Schmitz

Bassermannstr.1

68165 Mannheim

Phone 0621/4245575

Fax 0621/424-4807

The SUE form shall contain the following information:

Identification of the study patient, investigator in charge, description of the SUE (event, start and duration, outcome, causal relationship with the intervention, performed

treatment, date and signature of the supervising investigator)

Connection between intervention and Serious Adverse Event (SUE): O secured O probable O possible O unlikely O none O not assessable

Outcome of the Serious Adverse Event:

O Enhancement O Not Yet Restored O Permanent damage O Fatal , cause of death:

O unknown

In the event of an accumulation of or SUEs in a trial centre or trial arm (PTBD or EUCD), the Steering Committee is called in. The Steering Committee reserves the right to draw the appropriate consequences from the reports received, such as the closure of a trial site or termination of the trial.

**9. STATISTICAL METHODS**

In the following, the case number calculation and the statistical evaluation of the most important questions are described. Before the database is closed, the evaluation is defined in detail in a Statistical Analysis Plan (SAP).

9.1 Case Number Calculation

The calculation of the number of cases refers to the primary endpoint, the technical success rate. In the literature, rates of approximately 95% are given in similar studies (Lee et al. (2016)). This is assumed for both groups. If the technical success rate in the PTBD group is less than 10% lower than in the EUCD group, it is considered clinically irrelevant based on clinical judgment. Therefore, the non-inferiority threshold is defined as δ=0.1. At a unilateral significance level of 2.5%, 200 patients (100 per group) are required to perform a Farrington/Manning test at 80% power (calculations with PASS 14.0.8). It is assumed that for a very small proportion of patients the primary endpoint will not be met (for example, if a complication leads to premature discontinuation of the intervention). Therefore, at least 106 patients per group should be included to compensate for a loss of information due to 5% assumed drop-outs.

9.2 Statistical evaluation

The primary endpoint is the technical success rate in malignant distal choledochial stenosis using a self-expanding metal stent. The non-inferiority of PTBD compared to EUCD shall be demonstrated. The corresponding test hypothesis is

H0: pEUCD - pPTBD ≥ δ vs. H1: pEUCD - pPTBD < δ

Where pPTBD describes the technical success rate in the EUCD group, pPTBD the technical success rate in the PTBD group and δ=0.1 the non-inferiority limit.

The non-inferiority of PTBD to EUCD is verified by the Farrington/Manning test with a one-sided significance level of 2.5%.

Since this is a non-inferiority study, the primary evaluation will be based on both the Intention-To-Treat (ITT) Set and the Per-Protocol (PP) Set. In the ITT set, all patients are considered in whom the intervention was performed, even if it was technically unsuccessful or participation in the study was terminated prematurely. If the intervention has to be terminated prematurely (e.g. due to organ perforation), this patient is included in the primary evaluation. The primary evaluation is based on the assumption of technical failure in these patients.

In the PP Set, only those patients are included in the evaluation for whom the intervention was performed and the technical success was measured.

In sensitivity analyses, best case and worst case scenarios are calculated for the missing values. In addition, regression models are planned to investigate the influence of confounders on the endpoints. The variables intervention (EUCD with subgroups or PTBD with side localisation), BMI or bilirubin value are considered as input variables in the baseline study. The binary outcome variable is the technical success of the intervention. The affiliation of the individual patients to the two evaluation collectives is defined in SAP before the database is closed.

If non-inferiority can be shown with regard to the technical success rate, the occurrence of at least one complication (expected complication rate for both interventions: 10-20%) is compared hierarchically in the next step and tested for differences between the groups (based on the ITT set). Due to the hierarchical approach, the full level alpha, in this case two-sided 5%, is tested.

The evaluation of further secondary endpoints is done in the ITT set. With regard to the complication rate, PTCD is expected to have a lower complication rate than EUCD. No missing values are implanted during the evaluation of secondary endpoints. The evaluation is purely descriptive. Appropriate statistical methods are used according to the underlying distribution. For continuous endpoints, the mean, standard deviation, median, interquartile distance, and minimum and maximum are reported separately and in total for the two groups. For categorical endpoints, absolute and relative frequencies are given, also separately for the two groups and in total. Event times are displayed and evaluated using survival time analysis methods (e.g. Kaplan Meier). Descriptive p-values are given together with 95% confidence intervals. In the EUCD group, descriptive p-values are also divided by method (see 5.1). In addition, unless defined in the secondary endpoints, adverse events are evaluated and compared.

9.3 Interim analysis

No formal interim evaluation is planned.

The two interventions to be compared in this study have been in place for some time. Serious safety-relevant events are not expected to be observed. Therefore, no Data Safety and Monitoring Board (DSMB) will be established. The Steering Committee will decide on the continuation of the study based on the recruitment figures.

**10. DATA MANAGEMENT**

10.1 Documentation

All data collected within the framework of the study are documented on standardised survey forms (Case Report Forms = CRFs and reporting forms). The CRFs must ensure the complete documentation of all patient data to be collected according to the study protocol. They are therefore developed according to the guidelines of this study protocol by the Institute for Medical Biometry and Informatics (IMBI) Heidelberg in cooperation with the LKP.

The investigator is responsible for ensuring that all parts of the CRFs are filled out correctly. The CRFs are accompanied by filling instructions. These include instructions for documenting missing data.

According to ICH-GCP, errors in completing the CRFs should be crossed out by a single line so that the original entry remains legible, and the new entry should be dated and initialled. For self-explanatory corrections (e.g. twisted numbers in the date) the justification can be omitted. Each CRF must be signed at least once by the investigator. The completed original pages of the CRF are sent to the data management at the IMBI, a copy remains at the study centre.

10.2 Data management

Database development, data acquisition by means of double entry, data management and data validation are the responsibility of the study management in Mannheim. All data management processes are carried out according to Standard Operation Procedures (SOPs). Data validation includes checks for completeness, consistency and plausibility of the data documented in the CRF. For this purpose, a query system is established between data management and the investigator. In the so-called query process, queries are sent from data management to the trial centre as soon as possible to clarify incomplete, implausible and/or inconsistent data. These queries are answered by the investigator, or a person authorized by the investigator, and then sent to Data Management for entry into the database. After all queries for all included patients have been solved, the database is closed at the end of the study and handed over to the biometrician for evaluation. After completion of all evaluations and preparation of the final report, the originals of all CRFs are transferred to the LKP for archiving.

At the end of the study, the data are transformed into various data formats (e.g. csv files) to ensure further use. It is planned to make all primary data on which the scientific publications are based publicly available for re- and meta-analysis after the end of the study. During the course of the study, a suitable repository will be searched for and a decision will be made on this at the latest at the end of the study.

10.3 Storage and archiving of data

In accordance with the Medical Professional Code, all important study documents (e.g. CRFs) are archived for at least 10 years after the end of the examination. The chief clinical investigator (LKP) or his study assistant/study nurse is responsible for archiving.

The documents should contain Protocol, ethics committee application, ethics committee vote, patient information, informed consent form, final report, CRFs, and reporting forms.

Any change in ownership of the data is documented. All data will be made available to regulatory authorities upon request.

The investigators will archive all study data (source data) including patient identification list and relevant correspondence in accordance with Section 4.9 of the ICH Good Clinical Practice Guidelines (E6).

**11 ETHICAL AND LEGAL ASPECTS**

11.1 Good clinical practice

The procedures described for conducting, evaluating and documenting this clinical trial are intended to ensure that all parties involved adhere to the principles of Good Clinical Practice (GCP) and the ethical principles laid down in the Declaration of Helsinki.

The trial will be conducted in accordance with the "Basic Ethical Principles for Human Medical Research" as adopted by the 18th General Assembly of the World Medical Association in Helsinki (1964) with all subsequent amendments (until 2013). The applicable version for the respective country will be considered.

11.2 Ethics Committee Approval of Protocol and Amendments

Before the start of the clinical trial, the protocol, patient information and informed consent and all other necessary documents are submitted to the competent ethics committee (EC).

The approval of the ethics committee is a prerequisite for the start of the clinical trial. The opinion of the EC should include the title of the trial, the acronym if applicable, the trial sites, and any other documents reviewed. The date on which the decision was taken must be mentioned and the opinion must be signed by a member of the ethics committee. The documentation for the favourable evaluation is completed by a list of the members of the ethics committee who were involved in the consultation and a confirmation that the EC is working according to GCP principles (if necessary, the statutes of the EC can be filed together with the vote instead).

All correspondence (written and oral) with the responsible ethics committee must be documented and retained by the lead investigator.

All ethical and legal requirements must be met before the first trial subject is accepted into the clinical trial.

Changes to the protocol are made in writing and require the agreement of all signatories to the protocol. Subsequent substantial amendments to the protocol also require the approval of the Ethics Committee.

11.3 Practical Information and Consent of Study Participants

Before a trial subject can be enrolled in the trial, he or she must be informed by the investigator or an appropriately qualified member of the trial team about the nature, significance and individual scope of the trial, including the right to withdraw from the trial at any time, in an understandable form, both orally and in writing, and must subsequently consent to participation in writing. Consent must be obtained before the first intervention (PTBD or EUCD).

The trial subject should also have the opportunity to question the investigator or a medical member of the trial team about details of the trial. Withdrawal of consent may be given orally or in writing by the trial subject to the investigator-in-charge or another medical member of the study team at any time during the study. The trial subject should not be disadvantaged as a result,

and he must not be pressured into participating any longer. The patient is also not obliged to disclose the reasons for withdrawing consent.

After reading the explanatory letter and the declaration of consent, the patient must confirm his or her consent by signing it with his or her own handwritten and dated signature.

If the patient cannot read/write, the contents of the educational letter, the declaration of consent and the privacy statement must be presented and explained orally in the presence of an impartial witness. The witness and the doctor providing the information must then sign the documents personally and date them. The witness must not be dependent in any way on the principal investigator, the study site or any member of the study team (e.g., a study site employee).

The trial subject will receive a copy of the patient information and consent for the clinical trial. The original is kept by the investigator. These documents must be written in a language that the trial subject understands. They shall specify who informed the trial subject.

The trial subjects shall be informed of any new information that might influence their decision to participate in the trial. The communication of this information shall be documented.

How is the consent of the trial subject obtained? The investigator will obtain a voluntary written informed consent from each trial subject, after having adequately informed them of the objectives, methods, suspected benefits, potential risks, and any other aspect relevant to the decision to participate. The informed consent form must be signed and the name and date must be noted by the participant before the patient may be subjected to any trial-related procedures (including screening tests to determine suitability).

This study does not include persons who are not able to give consent.

**12 QUALITY CONTROL AND QUALITY ASSURANCE**

12.1 Data protection

The data collected in the course of the clinical trial are treated in accordance with the provisions of the Basic Data Protection Regulation (EU-DSGVO).

Accordingly, the patient has the right of access to the stored personal data relating to him/her (Art. 15 DS-GVO). If he or she should discover that incorrect personal data relating to him or her is being processed, he or she may request correction (Art. 16 DS-GVO). He has the right to request the deletion of his personal data if there are specific reasons for deletion. This is the case, for example, if the personal data are no longer necessary for the purpose for which they were originally collected or processed or if he withdraws his consent and there is no other legal basis for the processing (Art. 17 DS-GVO). In addition, he/she has the right to restrict the processing of his/her personal data (Art. 18 DS-GVO), to data transferability (Art. 20 DS-GVO) and a general right of objection (Art. 21 DS-GVO).

A data controller responsible for the trial centre is named in the patient information and consent form.

The patient has the right to complain to the supervisory authority if he/she believes that his/her personal data are not being processed lawfully. The address of the regulatory authority responsible for the trial site will be provided in the patient information and consent form.

During the clinical trial, participants are identified solely by means of an individual identification number (e.g. patient number). the data are treated in strict confidence. Any disclosure of this data to unauthorized third parties must be strictly prevented. The relevant provisions of country-specific data legislation are fully complied with.

With the written consent to participate in the clinical trial, the participant releases the investigator from his or her duty of confidentiality vis-à-vis representatives of the competent authorities (inspectors) and the sponsor (monitors, auditors) to the extent that these persons can inspect the personal data to verify the correct data transfer in order to check the proper conduct of the clinical trial.

The investigator is responsible for maintaining an identification list of trial subjects (subject's identification number and name) in order to enable identification if necessary.

12.2 Responsibilities of the investigator

The investigator shall ensure that all staff involved in the trial at the trial site are adequately informed about the protocol, any amendments to the protocol, trial treatments, and trial-related duties and tasks.

The investigator maintains a list of co-investigators and other qualified personnel to whom he or she has delegated important trial-related duties.

**13 AGREEMENTS**

13.1 Financing of the clinical trial

The clinical trial is financed by third-party funds from Theresienkrankenhaus and St. Hedwigsklinik GmbH, academic teaching hospital of the University of Heidelberg.

13.2 Reports

The responsible medical writer, in this case the chief clinical investigator (LKP), prepares a final study report in coordination with the investigators of the trial sites.

The study report is prepared with the support of the Institute for Medical Biometry and Informatics (Department of Medical Biometry) Im Neuenheimer Feld 130.3 in 69120 Heidelberg.

The following schedule is envisaged:

DBL (database lock): [30.06.2020]

Completion of statistical analysis: [1.10.2020]

Final study report: [1.12.2021]

13.3 Registration of the clinical trial

The lead investigator has registered the study with the study registry number: NCT03546049 at ClinicialTrials.gov.

13.4 Publication

The publication and/or presentation of the study results is expressly desired.

The study results are first communicated in confidence to all participating study centres and then submitted for publication in a peer-reviewed journal. The author(s) of the publication must be sufficiently authorised by the trial centres to publish the study results.

When publishing/presenting study results, no personal data of study participants may be disclosed that would enable their identification.

**References:**

1. Speer AG, Cotton PB, Russell RC, Mason RR, Hatfield AR, Leung JW, MacRae KD, Houghton J, Lennon CA. Randomised trial of endoscopic versus percutaneous stent insertion in malignant obstructive jaundice. Lancet. 1987 Jul 11;2(8550):57-62.

2. Giovannini M, Moutardier V, Pesenti C, Bories E, Lelong B, Delpero JR. Endoscopic ultrasound-guided bilioduodenal anastomosis: a new technique for biliary drainage. Endoscopy. 2001 Oct;33(10):898-900.

3. Sharaiha RZ, Khan MA, Kamal F, Tyberg A, Tombazzi CR, Ali B, Tombazzi C, Kahaleh

M. Efficacy and safety of EUS-guided biliary drainage in comparison with percutaneous biliary drainage when ERCP fails: a systematic review and meta-analysis. Gastrointest Endosc. 2017 May;85(5):904-914.

4. Bapaye A, Dubale N, Aher A. Comparison of endosonography-guided vs.percutaneous biliary stenting when papilla is inaccessible for ERCP. United European Gastroenterol J. 2013 Aug;1(4):285-93.

5. Artifon EL, Aparicio D, Paione JB, Lo SK, Bordini A, Rabello C, Otoch JP, Gupta K. Biliary drainage in patients with unresectable, malignant obstruction where ERCP fails: endoscopic ultrasonography-guided choledochoduodenostomy versus percutaneous drainage. J Clin Gastroenterol. 2012 Oct;46(9):768-74.

6. Khashab MA, Valeshabad AK, Afghani E, Singh VK, Kumbhari V, Messallam A,Saxena P, El Zein M, Lennon AM, Canto MI, Kalloo AN. A comparative evaluation of EUS-guided biliary drainage and percutaneous drainage in patients with distal malignant biliary obstruction and failed ERCP. Dig Dis Sci. 2015 Feb;60(2):557-65.

7. Sharaiha RZ, Kumta NA, Desai AP, DeFilippis EM, Gabr M, Sarkisian AM, Salgado S, Millman J, Benvenuto A, Cohen M, Tyberg A, Gaidhane M, Kahaleh M. Endoscopic ultrasound-guided biliary drainage versus percutaneous transhepatic biliary drainage: predictors of successful outcome in patients who fail endoscopic retrograde cholangiopancreatography. Surg Endosc. 2016 Dec;30(12):5500-5505.

8. Lee TH, Choi JH, Park do H, Song TJ, Kim DU, Paik WH, Hwangbo Y, Lee SS, Seo DW, Lee SK, Kim MH. Similar Efficacies of Endoscopic Ultrasound-guided Transmural and Percutaneous Drainage for Malignant Distal Biliary Obstruction. Clin Gastroenterol Hepatol. 2016 Jul;14(7):1011-1019.

9. Sportes A, Camus M, Greget M, Leblanc S, Coriat R, Hochberger J, Chaussade S, Grabar S, Prat F. Endoscopic ultrasound-guided hepaticogastrostomy versus percutaneous transhepatic drainage for malignant biliary obstruction after failed endoscopic retrograde cholangiopancreatography: a retrospective expertise-based study from two centers. Therap Adv Gastroenterol. 2017 Jun;10(6):483-493.

10. Schmitz D, Weller N, Doll M et al.. An improved method of percutaneous transhepatic biliary drainage combining ultrasound-guided bile duct puncture with metal stent implantation by fluoroscopic guidance and endoscopic visualization as a one step- procedure: a retrospective cohort (manuscript accepted in J Clin Int Rad 2018)

11. Schmitz D, Grosse A, Hallscheidt P, Roseneck A, Niemeyer J, Rudi J. ColorDoppler ultrasound-guided PTBD with and without metal stent implantation by endoscopic control: prospective success and early adverse event rates. Z Gastroenterol. 2015 Nov;53(11):1255-60.

12. Liu YS, Lin CY, Chuang MT, Tsai YS, Wang CK, Ou MC. Success and complications of percutaneous transhepatic biliary drainage are influenced by liver entry segment and level of catheter placement. Abdom Radiol (NY). 2017 Jul 24.

13. Shimizu H, Kato A, Takayashiki T et al..Peripheral portal vein-oriented non-dilated bile duct puncture for percutaneous transhepatic biliary drainage. World J Gastroenterol. 2015; 21(44)12628-12634.

14. Will U, Thieme A, Fueldner F, Gerlach R, Wanzar I, Meyer F. Treatment of biliary obstruction in selected patients by endoscopic ultrasonography (EUS)-guided transluminal biliary drainage. Endoscopy. 2007 Apr;39(4):292-5.

15. Tyberg A, Desai AP, Kumta NA, Brown E, Gaidhane M, Sharaiha RZ, Kahaleh M. EUS- guided biliary drainage after failed ERCP: a novel algorithm individualized based on patient anatomy. Gastrointest Endosc. 2016 Dec;84(6):941-946.

16. Cotton PB, Eisen GM, Aabakken L, Baron TH, Hutter MM, Jacobson BC, Mergener K, Nemcek A Jr, Petersen BT, Petrini JL, Pike IM, Rabeneck L, Romagnuolo J, Vargo JJ. A lexicon for endoscopic adverse events: report of an ASGE workshop. Gastrointest Endosc. 2010 Mar;71(3):446-54.

17. Saad WE, Wallace MJ, Wojak JC, Kundu S, Cardella JF. Quality improvement guidelines for percutaneous transhepatic cholangiography, biliary drainage, and percutaneous cholecystostomy. J Vasc Interv Radiol. 2010 Jun;21(6):789-95.

18. Gerbershagen HJ et al. W. Pain intensity on the first day after surgery: a prospective cohort study comparing 179 surgical procedures. Anesthesiology. 2013 Apr;118(4):934- 44.

19. Minaga K, Kitano M. Recent advances in endoscopic ultrasound-guided biliary drainage. Digestive Endoscopy 2017 Jun 28.
