# Supplementary material for: Percutaneous transhepatic or endoscopic ultrasound-guided biliary drainage in malignant distal bile duct obstruction using a self-expanding metal stent: Study protocol for a prospective European multicenter trial (PUMa trial)
Source: PLoS One. 2022 Oct 27;17(10):e0275029. doi: 10.1371/journal.pone.0275029 (PMC9612485; doi:10.1371/journal.pone.0275029)
Supplement: S4 File — (DOCX) [file pone.0275029.s004.docx]

1. The basic data protection regulation was implemented as the common data protection framework in the European Union since 25 May 2018 (EU-DSGVO). Therefore, relevant rules concerning the data protection of the study patients were redefined in chapter 12.1 (data protection).

2. Extension of the study centers from six centers in Germany to fourteen centers in Germany and Spain.

3. The use of a LAMS for EUS-CDS is explicitly allowed in chapter 5.1 (description of interventions).

4. PD Dr. Jochen Weigt was elected to the Steering Committee as Prof. U. Will has resigned.
