# Supplementary material for: Percutaneous transhepatic or endoscopic ultrasound-guided biliary drainage in malignant distal bile duct obstruction using a self-expanding metal stent: Study protocol for a prospective European multicenter trial (PUMa trial)
Source: PLoS One. 2022 Oct 27;17(10):e0275029. doi: 10.1371/journal.pone.0275029 (PMC9612485; doi:10.1371/journal.pone.0275029)
Supplement: S3 File — (DOCX) [file pone.0275029.s003.docx]

UMM University Medicine Mannheim

Institutional Review Board II , Theodor-Kutzer-Ufer 1-3, 68167 Mannheim

Chairman: Prof. Dr. med. Jens P. Striebel

Office: S. Cao, M. Goerner, K. Heberlein

Telephone: +49 621 383 - 9706 / - 9707 / - 9708 Telefax: +49 621 383 - 9710

ethikkommission-ll@medma.uni-heidelberg.de

[www.umm.uni-heidelberg.de/forschung/medizinische-ethikkommission](http://www.umm.uni-heidelberg.de/forschung/medizinische-ethikkommission) II

Mr.

Dr. Daniel Schmitz

Theresienkrankenhaus und St. Hedwigsklinik GmbH Mannheim

University of Heidelberg

Bassermannstr. 1

68165 Mannheim, Germany

Mannheim, 06.04.2018 / MG

**Our sign:	2018-522N-MA**

**Study title:**	Percutaneous transhepatic biliary drainage (PTBD) or endoscopic

ultrasound-guided biliary drainage (EUS-BD) with metal stent implantation in

malignant distal bile duct obstruction: study protocol for a prospective European

multicentre trial (**PUMa-Study**)

**Study Director:**	Dr. med. Daniel Schmitz

**Leading Study Centre:**	Theresienkrankenhaus	und	St. Hedwigsklinik	GmbH, University of

Heidelberg, Dep. of Gastroenterology, Oncology and Diabetology,

Bassermannstr. 1, 68165 Mannheim, received 07.02.2018

**Ethical approval**

Dear Dr. Schmitz,

the Institutional Review Board II of the Medical Faculty Mannheim of the University of Heidelberg has approved the above-mentioned research project under consideration of the following documents:

- ethical approval of 22.01.2018
- patient information and consent form, version 1.0 from 04.11.2017
- study protocol, version 3.1 of 21.01.2018

Requested or accepted modifications, received on 05.04.2018

- Statement of 10.03.2018
- Patient information and declaration of consent, version 1.1 of 10.03.2018
- Study protocol, version 3.2 of 10.03.2018

The members of the Institutional Review Board II listed below have discussed the application of ethical approval on 20.02.2018 and came to following decision:

**There are no objections to the study project**

**from an ethical and professional point of view.**

The following notes and conditions apply to this ethical approval:

1. Please also note the changes required in the chapter on data protection due to the EU-DSGVO which will come into force on 25 March 2018.
2. The Institutional Review Board points out that the information of the patient on the study project must always be done orally, as the exclusive use of information forms does not address the specific circumstances of each study participant. The signed, handwritten information form and consent is only intended to relieve the medical director of the potential accusation of inadequate information, since he bears the burden of proof for adequate information.
3. The Institutional Review Board must receive information on serious or unexpected unwanted adverse events occurring during the study that could affect the safety of the study participants or the conduct of the study. A reasoned statement of the study director has to be added together with a note whether, in his view, the benefit/risk ratio of the project has changed.
4. Ethically relevant changes in the study protocol and in the course of study must be notified to the Institutional Review Board and changes in the documents should be clearly highlighted.
5. The applicant must ensure that the files and documents of the study are stored at his

current field of work and managed in an orderly manner after completion of the study .

6. The Institutional Review Board points out that the use of this study approval is linked to the obligation that the Institutional Review Board II has to be informed on the results of the study within 6 months after completion of the study (report summarising the study results or original publication if necessary).

This decision was taken unanimously.

Regardless of the result of the consultation of the Institutional Review Board II, I would like to point out that the ethical and legal responsibility for the conduct of a clinical trial lies with the study director of the clinical

trial and also with all physicians participating in the trial.

The Institutional Review Board II of the University of Heidelberg at the Mannheim Medical Faculty works according to national legal regulations and the ICH-GCP guidelines. The consultations of the Institutional Review Board II are based on the authoritative Declaration of the World Medical Association of Helsinki in the revised version of 2013.

With kind regards,

Members of the Institutional Review Board II:

Dr. med. Dorothee Darr, M.A.

Specialist in anaesthesiology, consultant for clinical ethics, University Medicine Mannheim

Prof. Dr. med. Lutz Frölich, (Deputy Chairman)

Head of the Department of Gerontopsychiatry and the dementia competence network, Clinic for Psychiatry and Psychotherapy at the Central Institute for Mental Health Mannheim, Medical Faculty Mannheim, University of Heidelberg

Prof. Dr. iur. Stefan J. Geibel

Maitre en droit (Universite Aix-Marseille III)

Institute for German and European Corporate and Commercial Law, University of Heidelberg

Dorothea Gulba, M. A.

Clinical Pastor, Master of Supervision, University Medicine Mannheim

Prof. Dr. med. Hans-Peter Hammes

Doctor of internal medicine, endocrinology and diabetology, head of Department of Endocrinology,

V. Medical Clinic, University Medicine Mannheim

Prof. (apl.) Dr. Dieter Kleinbohl

Dr. phil., graduate psychologist, Institute for Neuropsychology and Clinical Psychology

at the Central Institute for Mental Health Mannheim, Medical Faculty Mannheim of the University of Heidelberg

Helga Kühlwein, M. A.

Certified pedagogue, elected representative of severely disabled people, University Medicine Mannheim

Dr. rer. nat. Christine Lorenz

Pharmacist, Pharmacy Service Centre, University Medicine Mannheim

PD Dr. med. Eva Neumaier-Probst

Specialist in radiology and neuroradiology, Department of Neuroradiology, University Medicine Mannheim

Prof. Dr. med. vet. Gisbert Sponer

Professor of Pharmacology and Toxicology, Medical Faculty Mannheim of the University of Heidelberg

Prof. Dr. med. Jens-Peter Striebel, (Chairman)

Apl. Professor of Anaesthesiology and Intensive Care Medicine, Emergency Medicine, Pain Therapy and Blood Transfusion, Mannheim Medical Faculty of Heidelberg University

Prof. Dr. med. Karl-Ludwig Waag, (2nd vice chairman)

Apl. Full Professor of Pediatric Surgery and Specialist in Surgery, Medical Faculty Mannheim of the University of Heidelberg

Prof. Dr. sc. Hum. Christel Weil

Apl. Prof. for Biomathematics and Epidemiology, Diplom-Mathematician, Department of Medical Statistics, Biomathematics and Information Processing, Medical Faculty Mannheim of Heidelberg University

UMM University Medicine Mannheim

Institutional Review Board II , Theodor-Kutzer-Ufer 1-3, 68167 Mannheim

Chairman: Prof. Dr. med. Jens P. Striebel

Office: S. Cao, M. Goerner, K. Heberlein

Telephone: +49 621 383 - 9706 / - 9707 / - 9708 Telefax: +49 621 383 - 9710

ethikkommission-ll@medma.uni-heidelberg.de

[www.umm.uni-heidelberg.de/forschung/medizinische-ethikkommission](http://www.umm.uni-heidelberg.de/forschung/medizinische-ethikkommission) II

Mr.

Dr. Daniel Schmitz

Theresienkrankenhaus und St. Hedwigsklinik GmbH Mannheim

University of Heidelberg

Bassermannstr. 1

68165 Mannheim, Germany

Mannheim, 13.08.2019 / MG

**Our sign:	2018-522N-MA**

**Study title:**	Percutaneous transhepatic biliary drainage (PTBD) or endoscopic

ultrasound-guided biliary drainage (EUS-BD) with metal stent implantation in

malignant distal bile duct obstruction: study protocol for a prospective European

multicentre trial (**PUMa-Study**)

**Study Director:**	Dr. med. Daniel Schmitz

**Leading Study Centre:**	Theresienkrankenhaus	und	St. Hedwigsklinik	GmbH, University of

Heidelberg, Dep. of Gastroenterology, Oncology and Diabetology,

Bassermannstr. 1, 68165 Mannheim, received 07.02.2018

Amendment from 09.08.2019:

- Trial protocol, version 4.3 from 08.08.2019
- Patient information and consent form, version 2.3 from 08.08.2019Dear Dr Schmitz,

Dear Dr. Schmitz,

thank you very much for the submitted documents.

After reviewing the documents, the Medical Ethics Committee II is of the opinion that the notified changes are in accordance with the positive vote of the Ethics Committee II of 06.04.2018.

There are no ethical or professional objections to the further conduct of the above-mentioned study.

Aspects of data protection law in research projects are generally only examined in a cursory manner by the Ethics Committee. This vote therefore does not replace consultation with the responsible company or official data protection officer.

With kind regards,

Theodor-Kutzer-Ufer 1-3, 68167 Mannheim, Germany. Register Court: Mannheim Local Court, HRB Mannheim 7331 Chairman of the Supervisory Board: Lord Mayor Dr. Peter Kurz Managing Directors: Freddy Bergmann, Prof. Dr. med. Hans-Jürgen Hennes

Mannheim Medical Faculty of the University of Heidelberg. Theodor-Kutzer-Ufer 1-3, 68167 Mannheim Dean: Prof. Dr. Sergij Goerdt
