# Supplementary material for: Percutaneous transhepatic or endoscopic ultrasound-guided biliary drainage in malignant distal bile duct obstruction using a self-expanding metal stent: Study protocol for a prospective European multicenter trial (PUMa trial)
Source: PLoS One. 2022 Oct 27;17(10):e0275029. doi: 10.1371/journal.pone.0275029 (PMC9612485; doi:10.1371/journal.pone.0275029)
Supplement: Attachment — Submitted filename: Response to reviewers.docx [file pone.0275029.s006.docx]

July 25, 2022

**Response to reviewers’ comments on manuscript PONE-D-21-38739**

Dear Lorena Verduci, Staff Editor of PLOS ONE,

thank you that our manuscript PONE-D-21-38739 was commented by your reviewers and considered for revision.

The revised manuscript with track changes uses blue for reviewer 1, red for reviewer 2 and green for reviewer 3.

In the following, we would like to respond to the reviewers´ comments in detail.

**Response to the requested journal requirements**

1. The missing PLOS ONE's style requirements were added and the whole manuscript corrected
2. The study investigators decided to omit the sentence “This protocol, the CRFs, and other study documents and materials must be treated with the highest degree of confidentiality and may not be disclosed to third parties unless the principal gives his express consent. Employees of the investigator are also bound by this agreement” in the original protocol. Furthermore, figure 1 was omitted as this is a figure with copyright (courtesy from Prof. Will). The cleaned protocol is uploaded as S2_Complete Study Protocol
3. A redundant ethics statement was deleted in the section “Amendments”
4. The reference to Figure 4 was deleted as no longer needed

**Response to the specific reviewers‘ comments:**

**Reviewer 1:**

1. Comments like "appropriate statistical methods ...." (see Statistical analysis section) should be avoided; clearly specify that you maybe using 2-group t-tests for variables that are Gaussian, and may resort to nonparametric analysis (such as, Wilcoxon rank sum test)

**Answer:** The sentence in the Statistical Analysis section was specified to:

Two group t-tests might be used for variables that are Gaussian. If necessary, the analysis might resort to nonparametric analysis such as Wilcoxon rank sum test

(Line 377-379)

2. Since this will be a non-randomized design, what will be the corresponding

CONSORT statements/guidelines to present? That statement is missing in this Study

Protocol

**Answer:** Reasons for the study design as a non-randomized study are given in the

section “Limitations”. Inclusion process will be reported in an adapted

CONSORT flow diagram as specified in the section “Follow up”. The sentence was

revised to:

Excluded patients and reasons for exclusion will be presented in an adapted

CONSORT flow diagram [19] (Line 273-274).

3. Clearly, owing to the multi-center nature of the proposal, I was wondering why the authors didn't include analysis plan that "controls" for this center clustering effect.
   **Answer:**

The concept of a non-randomized prospective study was chosen for this research to avoid a center clustering effect. However, logistic regression models might be used in the statistical analysis if necessary.

4. Survival analysis plan includes very basic assessments using Kaplan-Meier plots. Why isn't a regression plan (say, via Cox models, or alternative models when PH assumption is not satisfied) not part of the protocol, if a variety of other covariables can be collected during the study?

**Answer:**

Text was changed to:

Logistic regression models might be used for secondary endpoints including

propensity score matching if necessary (Line 384-386).

**Reviewer 2:**

1. As authors mention, non-randomized study is the limitation in this study. I recommend authors to describe how to overcome this limitation. For example, propensity score matching, or logistic regression analysis can be employed.

**Answer:**

Text was changed to:

Logistic regression models might be used for secondary endpoints including

propensity score matching if necessary (Line 384-386)

**Reviewer 3:**

1. Being biased background since this study will include gastric cancers and hepatobiliary malignancy that developed distal bile duct obstruction, it should be mentioned as one of the limitations of this study. Biological behavior of gastric cancers is quite different from that of hepato-biliary cancers.

**Answer:**

We agree to this comment. Survival after successful technical and clinical biliary

drainage will be affected by tumor entity and other confounding factors such as

medical oncological therapy. Therefore, this issue was added to the section

“Limitations”:

And finally, follow up-analysis of the treated patients might be confounded by the

underlying tumor entity and the received palliative medical oncological treatment.

(line 434-436)

2. Concerning the location of malignant biliary obstruction, please define the distal biliary obstruction for reader to understand. Would it be difficult to make a distinction difference between the distal and proximal?

**Answer:**

We specified the distinction between proximal and distal extrahepatic bile duct obstruction by adding a further item to the exclusion criteria:

**Proximal malignant extrahepatic bile duct obstruction defined as obstruction at the**

**level of the bifurcation of the extrahepatic bile duct and proximal of it (line 212-213)**

With kind regards,

Daniel Schmitz

PS: Please use the following email address for future correspondence: Daniel.Schmitz@helios-gesundheit.de
