# Supplementary material for: Percutaneous transhepatic or endoscopic ultrasound-guided biliary drainage in malignant distal bile duct obstruction using a self-expanding metal stent: Study protocol for a prospective European multicenter trial (PUMa trial)
Source: PLoS One. 2022 Oct 27;17(10):e0275029. doi: 10.1371/journal.pone.0275029 (PMC9612485; doi:10.1371/journal.pone.0275029)
Supplement: Attachment — Submitted filename: Response to reviewers.docx [file pone.0275029.s007.docx]

29 August 2022

**PONE-D-21-38739R1**

Percutaneous transhepatic or endoscopic ultrasound-guided biliary drainage in malignant distal bile duct obstruction using a self-expanding metal stent: Study protocol for a prospective European multicenter trial (PUMa trial)

PLOS ONE

**Response to reviewers**

I. **Journal Requirements:**

The reference list was reviewed to ensure that it is complete and correct. All cited papers are complete and correct.

Felix Grassmann from the Institute of Medical Statistics and Epidemiology was newly added to the author list as he substantially contributed to the statistical concept of the study protocol

II. **Response to Reviewers' comments:**

Have the authors described where all data underlying the findings will be made available when the study is complete?

The PLOS Data policy requires authors to make all data underlying the findings described in their manuscript fully available without restriction, with rare exception, at the time of publication. The data should be provided as part of the manuscript or its supporting information, or deposited to a public repository. For example, in addition to summary statistics, the data points behind means, medians and variance measures should be available. If there are restrictions on publicly sharing data—e.g. participant privacy or use of data from a third party—those must be specified.

Reviewer #1: No

Reviewer #2: Yes

Reviewer #3: Yes

Response:

The complete study protocol and all study results will be available in the public online repository (FAIRsharing ID 4350; https://fairsharing.org). The manuscript text was changed on page 13.

Review Comments to the Author

Reviewer #1: The authors addressed my questions, except one.

I asked: Survival analysis plan includes very basic assessments using Kaplan-Meier plots.

Why isn't a regression plan (say, via Cox models, or alternative models when PH

assumption is not satisfied) not part of the protocol, if a variety of other covariables can

be collected during the study?

Authors replied: Text was changed to:

Logistic regression models might be used for secondary endpoints including

propensity score matching if necessary (Line 384-386).

I have no clue what the authors meant here. If one has survival/time-to-event endpoints, one would like to do a Cox regression; logistic regression is a separate stuff. Please clarify, or make the appropriate correction.

Reviewer #2: Authors properly revised the manuscript according to my comments. I do not have any additional comments on it.

Reviewer #3: I have carefully read the revised version of this paper. The authors perfectly responded to all my concerns. I have no further comments.

________________________________________

7. PLOS authors have the option to publish the peer review history of their article (what does this mean?). If published, this will include your full peer review and any attached files.

If you choose “no”, your identity will remain anonymous but your review may still be made public.

Do you want your identity to be public for this peer review? For information about this choice, including consent withdrawal, please see our Privacy Policy.

Reviewer #1: No

Reviewer #2: No

Reviewer #3: Yes: Kosuke Minaga
